# phylosignal: an R package to measure, test, and explore the phylogenetic signal

**DOI:** 10.1002/ece3.2051

**Published:** 2016-03-19

**Authors:** François Keck, Frédéric Rimet, Agnès Bouchez, Alain Franc

**Affiliations:** ^1^ UMR CARRTELInstitut National de la Recherche Agronomique (INRA)F‐74203ThononFrance; ^2^ UMR CARRTELUniversité Savoie Mont‐BlancF‐73011ChambéryFrance; ^3^ UMR BIOGECOInstitut National de la Recherche Agronomique (INRA)F‐33610CestasFrance

**Keywords:** Autocorrelation, comparative analysis, phylogenetic correlogram, phylogenetic signal, R software, trait evolution

## Abstract

Phylogenetic signal is the tendency for closely related species to display similar trait values as a consequence of their phylogenetic proximity. Ecologists and evolutionary biologists are becoming increasingly interested in studying the phylogenetic signal and the processes which drive patterns of trait values in the phylogeny. Here, we present a new R package, phylosignal which provides a collection of tools to explore the phylogenetic signal for continuous biological traits. These tools are mainly based on the concept of autocorrelation and have been first developed in the field of spatial statistics. To illustrate the use of the package, we analyze the phylogenetic signal in pollution sensitivity for 17 species of diatoms.

## Introduction

A common observation is that continuous traits of closely related species in a phylogeny are often similar, especially when traits are under selection pressure of the environment. More generally, inheritance of traits passed with modifications from one generation to the next may lead to a structured repartition of trait values throughout the phylogeny. The link between phylogeny and continuous trait values is commonly referred in the literature as phylogenetic signal. This concept has gained in popularity among ecologists in recent years, but is often misunderstood and confused with other fundamental ideas like phylogenetic conservatism (Losos 2008). To avoid any possible confusion (see Revell et al. 2008 for disentangling both notions), we stick here to the strict statistical definition of the phylogenetic signal given by Blomberg and Garland (2002), that is, the “tendency for related species to resemble each other more than they resemble species drawn at random from the tree”. Thus, the phylogenetic signal is a statistical dependence between the values of a continuous trait and the phylogenetic tree from which the measured species are the leaves. Studying a statistical dependence leads to hypothesis testing, and formalizing a null hypothesis. Thus, the presence of phylogenetic signal (as defined by Blomberg & Garland) can be tested by rejecting the null hypothesis that trait values for two species are distributed independently from their phylogenetic distance in the tree.

The detection and correction of phylogenetic signal has long been motivated by the necessity to control for nonindependence of traits data in comparative studies (Felsenstein 1985; Abouheif 1999). However, recent works have shown that studying the phylogenetic signal can raise interesting biological and ecological perspectives. For example, deciphering the phylogenetic signal may help to understand community assembly processes (Webb et al. 2002), detect niche conservatism (Losos 2008), or identify evolutionary strategies (Jombart et al. 2010b).

There are two contrasting approaches in the way phylogenetic signal for a trait can be studied as a statistical model. The first one is based on an explicit evolutionary model for the trait. This is generally a Brownian motion model (Pagel 1999; Blomberg et al. 2003) where continuous traits evolve randomly over time along a branch, with a fixed rate. As soon as descents split at a node of the phylogeny, evolution on both branches becomes independent. To test the presence of phylogenetic signal, the null hypothesis is that trait values are randomly distributed in the phylogeny. Another null hypothesis might be that trait values follow a Brownian motion model but it is less often used and implemented. The second approach relates to methods based on the concept of autocorrelation, the correlation of a vector with itself for a given lag. Autocorrelation is a mathematical tool which has been extensively used to study spatial and time series data. They are designed to detect whether the location of an individual gives information on the expected values of its traits. However, these methods do not rely on any evolutionary model. In a phylogenetic context, patterns of trait values of the species of a tree can be framed as the outcome of a marked point process. Thus, phylogenetic tools based on autocorrelation were largely imported from spatial statistics (Cheverud et al. 1985; Gittleman and Kot 1990; Jombart et al. 2010b).

We present a new R package, phylosignal, designed to quantify the phylogenetic signal for continuous biological traits. Most of the tools implemented in phylosignal are based on the concept of autocorrelation and thus are imported from spatial statistics. As such, they are well documented and understood. In this paper, we show how they can be used in a phylogenetic context and we describe their implementation in the package. To illustrate the features of the package, we analyze the phylogenetic signal in pollution sensitivity for 17 species of diatoms.

## The phylosignal Package

The phylosignal package provides a collection of tools to visualize, measure, test, and explore the phylogenetic signal in continuous traits (Table 1). The package is written in R and C++ languages and is fully accessible through the R environment. The latest stable version is accessible from *The Comprehensive R Archive Network* (https://cran.r-project.org/web/packages/phylosignal/) while the development version is hosted on *GitHub* (https://github.com/fkeck/phylosignal). The phylosignal package is a free software released under the GNU GPL‐3 license and any contribution is welcome.

**Table 1:** List of the phylosignal package main functions and their description

Function	Description
barplot.phylo4d dotplot.phylo4d gridplot.phylo4d	Plots trait values along a phylogeny
phyloSignal	Computes and tests the phylogenetic signal with different methods
phyloSim plot.phyloSim	Simulations, to investigate the behavior of different phylogenetic signal statistics for a given phylogenetic tree along a gradient of signal
phyloSignalBS	Computes and plots phylogenetic signal for bootstrapped replicates of a phylogeny.
phyloSignalINT	Computes and tests the phylogenetic signal at each internal node of a phylogeny
phyloCorrelogram plot.phylocorrelogram	Computes and plots a phylogenetic correlogram or a multivariate Mantel correlogram
lipaMoran	Computes Local Indicator of Phylogenetic Association (local Moran's I)
graphClust plot.graphclust	Extracts clusters of species based on trait values and phylogenetic proximities
focusTree focusTraits focusTips focusStop	Utility functions to add graphical elements to plots created with barplot.phylo4d, dotplot.phylo4d, gridplot.phylo4d
phyloWeights	Utility function to compute a matrix of phylogenetic weights with different methods

This package builds on the R ecosystem richness and takes full advantage of ape (Paradis et al. 2004) for tree manipulation and plotting capacities and adephylo (Jombart et al. 2010a) for tree walking algorithms and phylogenetic distances computing.

### Data format

The analysis of phylogenetic signal typically involves working with a phylogeny and trait values associated with each tip (leaf). The phylobase package (Hackathon et al. 2013) defines the S4 class phylo4d designed specifically to handle such kind of data. Thus, a phylo4d object connects a phylogenetic tree with a table of trait values and constitutes the basic input for many functions implemented in phylosignal. The phylobase package comes with all the necessary functions to construct and manipulate phylo4d objects. For the users who are not used to handle phylogenetic data within the R environment, phylosignal adds the simple function read.p4d, which constructs a phylo4d object from a phylogenetic tree stored in a Newick file and tips data stored in a CSV file.

### Data visualization

**Figure 1:**
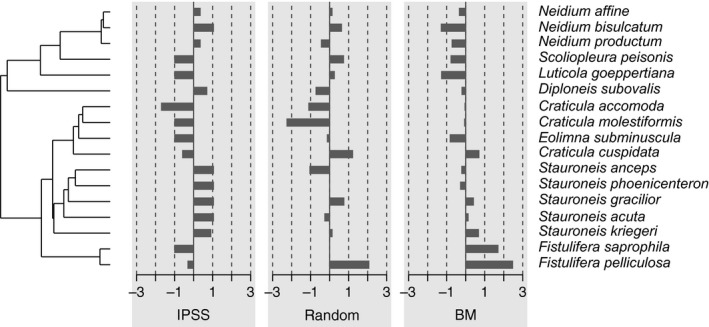
Data visualization of 3 traits (IPSS, random, BM) mapped along the phylogeny of 17 diatom species. This output is obtained with the function barplot.phylo4d. By default data are centered and scaled by trait.

The first step of any statistical analysis should be a graphical exploration of the data. The R language provides very powerful and flexible graphics facilities (Murrell 2005). They are extended for phylogenetic tree visualization with traits data by many packages: ape (Paradis et al. 2004), phytools (Revell 2012), adephylo (Jombart et al. 2010a). The phylosignal package aims to provide a simple but complete interface to map traits data onto a phylogenetic tree. The users have access to three main functions to generate high quality graphics: barplot.phylo4d, dotplot.phylo4d and gridplot.phylo4d, which can, respectively, represent univariate and multivariate traits data as bars, dots, and colored cells. Each of these functions comes with several arguments to precisely control graphical aspects. Figure 1 gives an example of a graphic generated with barplot.phylo4d.

### Indices for general measurements of phylogenetic signal

The function phyloSignal provides a generic interface to compute indices and tests on multiple traits from a phylo4d object. The package implements two methods directly based on the autocorrelation principle.

- The Moran's I index (Moran 1948, 1950) is the standard measure of autocorrelation used in spatial statistics and has been proposed as a way to measure the phylogenetic signal by Gittleman and Kot (1990). The function phyloSignal computes I using Equation (1) with *y*_*i*_ and *y*_*j*_ being the trait value measured for species *i* and species *j*, respectively, *n* being the number of species and, by default, $w_{ij}=\frac{1}{d_{ij}},d_{ij}$ being the patristic distance between species *i* and species *j*.

(1) $$I=\frac{n}{\sum_{i=1}^{n}\sum_{j=1}^{n}w_{ij}}\frac{\sum_{i=1}^{n}\sum_{j=1}^{n}w_{ij}\left(y_{i}-\overset{¯}{y}\right)\left(y_{j}-\overset{¯}{y}\right)}{\sum_{i=1}^{n}{\left(y_{i}-\overset{¯}{y}\right)}^{2}}$$

- The Abouheif's *C*_mean_ index (Abouheif 1999) has been shown to be a Moran's I index computed with a specific matrix of phylogenetic weights (Pavoine et al. 2008). Thus, phyloSignal computes *C*_mean_ using Equation (1) with w_*ij*_ being the proximity matrix A described in Pavoine et al. (2008) and computed with proxTips(x, method = ”Abouheif”) from adephylo.

Additionally, the function phyloSignal can compute three indices based on evolutionary models: Blomberg's K and K* (Blomberg et al. 2003) and Pagel's λ (Pagel 1999).

Each index can be tested for the null hypothesis of absence of signal (i.e., trait values are randomly distributed in the phylogeny). This is achieved by randomization for K, K*, *C*_mean_, and I and by likelihood ratio test for λ. Indices and tests procedures are written in C++ to optimize speed when dealing with large phylogenies, multiple traits, and simulations.

Choosing an appropriate method to measure and test the phylogenetic signal is not straightforward. Münkemüller et al. (2012) provided general and useful guidelines, but stress that the behavior of indices strongly depends on numerous parameters like phylogenetic tree topology, sample size, and complexity of the evolutionary models generating traits patterns. Moreover, phylogenetic trees based on real data can differ greatly from simulated trees commonly used in simulations. Therefore, it can be interesting to investigate how the indices behave with the phylogeny under study. The phyloSim function takes up the method described by Münkemüller et al. (2012) to simulate traits with variable strength of Brownian motion for a given phylogeny and then computes indices and tests along a gradient of phylogenetic signal. Results of these simulations can be used to compare the performances of the different methods and interpret indices' values obtained with real traits data, for a given phylogeny.

### The phylogenetic correlogram

The phylogenetic correlogram takes up the core idea of the spatial correlogram (Sokal and Oden 1978). It aims to graphically represent how the data are autocorrelated at different lags of distance. The idea was introduced in a phylogenetic context by Gittleman and Kot (1990) as a way to locate the phylogenetic signal in the taxonomy. Using an accurate phylogeny, it is possible to replace taxonomic distances with phylogenetic distances (e.g., patristic distance). This method has been promoted by Hardy and Pavoine (2012) as an interesting way to characterize the nature of the phylogenetic signal especially when model‐based approaches are limited by the complexity of evolutionary processes.

However, an inherent issue of correlograms is that the autocorrelation must be computed within discretized distance classes. Therefore, the use of the correlogram may be strongly limited for small trees and when tips are not uniformly distributed within the phylogeny. In response to this potential problem, the phylosignal package comes with an original implementation of the phylogenetic correlogram for which the autocorrelation can be computed continuously. This is achieved by computing the Moran's I index using a specific matrix of phylogenetic weights w based on a normalized Gaussian function (Equation (2)).

(2) $$w_{ij}=\frac{1}{\sigma \sqrt{2\pi }}e^{\frac{{\left(d_{ij}-\mu \right)}^{2}}{2\sigma^{2}}}$$

**Figure 2:**
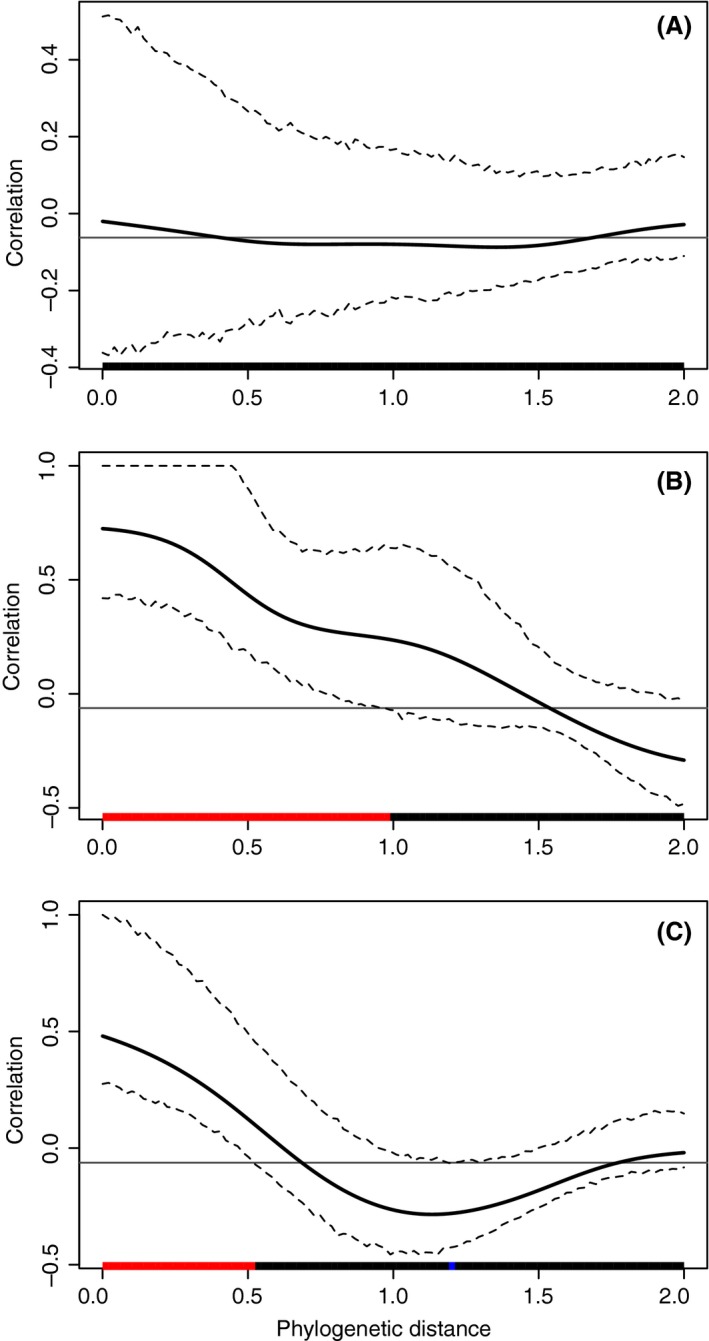
Phylogenetic correlograms for 3 traits: (A) *random*, (B) *BM*, and (C) *IPSS*. The solid bold black line represents the Moran's I index of autocorrelation, and the dashed black lines represent the lower and upper bounds of the confidence envelop (here 95%). The horizontal black line indicates the expected value of Moran's I under the null hypothesis of no phylogenetic autocorrelation. The colored bar show whether the autocorrelation is significant (based on the confidence interval): red for significant positive autocorrelation, black for nonsignificant autocorrelation, and blue for significant negative autocorrelation.

Therefore, a phylogenetic weight matrix can be computed giving μ, which defines the distance at which a tip will have the strongest influence and σ which defines the decrease of influence around μ. This matrix can be computed using the function phyloWeights, but the phylogenetic correlogram can be estimated directly with the function phyloCorrelogram. Additionally, a confidence envelope is computed using nonparametric bootstrap resampling. Finally, the function can estimate a multivariate Mantel correlogram (Oden and Sokal 1986) if two traits or more are provided. Figure 2 gives an example of phylogenetic correlograms with their confidence envelope.

### Local Indicators of Phylogenetic Association

Global measurement of autocorrelation like Moran's I and phylogenetic autocorrelograms gives precious information about the general presence of a phylogenetic signal within a phylogeny. However, these approaches make the implicit assumptions that traits evolve similarly across the phylogeny. There are solid grounds to expect that this is rarely the case and that phylogenetic signal is scale dependent and varies among clades. Therefore, it can be interesting to use local statistics to describe local traits patterns.

Spatial statistics have introduced a class of statistical tools to analyze local patterns called *Local Indicators of Spatial Association* (LISA). One simple and well‐described LISA is the local Moran's I (Equation (3)), noted I_*i*_ (Anselin 1995), which can be used to detect hotspots of positive and negative autocorrelation. The same statistic can be applied to phylogenetic data to detect species with similar neighbors and species with different neighbors. In this context, we call these indicators *Local Indicators of Phylogenetic Association* (LIPA), for sake of consistency in terminology, although the statistic remains the same.

(3) $$I_{i}=\frac{y_{i}-\overset{¯}{y}}{m_{2}}\sum_{j=1}^{n}w_{ij}\left(y_{j}-\overset{¯}{y}\right)$$

With

$$m_{2}=\frac{\sum_{i=1}^{n}{\left(y_{i}-\overset{¯}{y}\right)}^{2}}{n}$$

**Figure 3:**
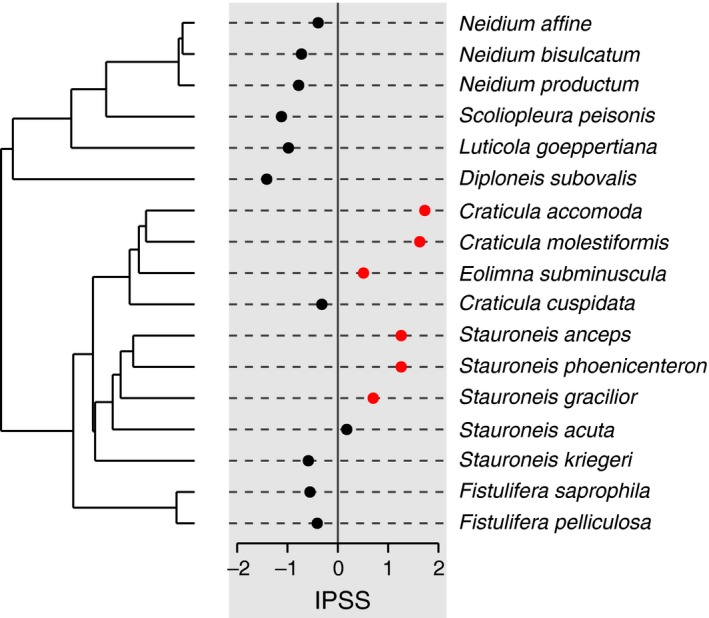
Local Moran's index (I_*i*_) values for each species for trait IPSS computed with lipaMoran and plotted with dotplot.phylo4d. Red points indicate significant I_*i*_ values.

Local Moran's I (I_*i*_) can be computed with the function lipaMoran for each tip of the phylogeny and for one or more traits. By default, the function uses a phylogenetic weights matrix $w_{ij}=\frac{1}{d_{ij}}$, *d*_*ij*_ being the patristic distance matrix. However, any matrix of weights can be provided. For each value of local Moran, the function performs a nonparametric test by randomization and returns a *P*‐value. Figure 3 gives an example of Local Moran's I (I_*i*_) values plotted onto a phylogenetic tree.

### Additional functionalities

The phylosignal package comes with some additional features to analyze phylogenetic signal. The function phyloSignalINT computes phylogenetic signal indices and tests for each internal node of a given phylogeny. Combined with lipaMoran, it can be helpful to identify an interesting region, exhibiting strong conservation, for example, in the phylogenetic tree. If bootstrapped replicates of the phylogeny are available, the function phyloSignalBS can be used to compute signal indices and tests for each bootstrap. The function renders the results as boxplots allowing assessing the effect of phylogenetic reconstruction uncertainty on phylogenetic signal estimates. Finally, the function graphClust implements a simple method to perform traits clustering under phylogenetic constraints (Keck et al. In press a).

## Example: Phylogenetic Signal of Pollution Sensitivity in Diatoms

In order to demonstrate the application of phylosignal, we comment on an analysis of the phylogenetic signal for 17 diatoms species. The trait analyzed is the specific pollution sensitivity index, *IPSS* (Coste 1982). The diatoms are taken from the order *Naviculales* and the phylogenetic tree is taken from Keck et al. (In press b). This dataset is deliberately kept simple for demonstration purposes: this is a very brief overview of the diversity existing in this clade but it constitutes a good case study (for a more comprehensive discussion about phylogenetic signal in diatoms sensitivity to pollutions, see Keck et al. In press a,b). The dataset is included in the package and can be loaded with the following command.



data(navic)




For illustration purposes, we add two other traits: *random* which is randomly distributed in the phylogeny and *BM* which is generated under a Brownian motion model.



library(ape)
library(phylobase)
tipData(navic)$random <‐ rnorm(17)
tipData(navic)$BM <‐ rTraitCont(as(navic, "phylo"))




The data are loaded in the form of a phylo4d object. It is therefore extremely easy to plot the phylogeny and the trait values (Fig. 1).



barplot.phylo4d(navic)




We can compute phylogenetic signal indices and *P*‐values of their respective tests.



phyloSignal(navic)






$stat
 Cmean I K
IPSS 0.47915189 0.04286040 0.7897245
random −0.06522342 −0.10555838 0.3213491
BM 0.37543446 0.08060191 0.7267358
 K.star Lambda
IPSS 0.8541988 0.9588398276
random 0.3216638 0.0000704802
BM 0.7852155 0.9798037571






$pvalue
 Cmean I K K.star Lambda
IPSS 0.008 0.088 0.014 0.012 0.02593566
random 0.464 0.713 0.565 0.629 1.00000000
BM 0.006 0.035 0.014 0.008 0.07076068




Not surprisingly, tests tend to detect a signal for *BM* and not for *random*. The phylogenetic signal also appears to be significant for *IPSS*. We can compute and plot a phylogenetic correlogram for each trait with the following commands:



IPSS.cg <‐ phyloCorrelogram(navic, trait = "IPSS")
random.cg <‐ phyloCorrelogram(navic, trait = "random">)
BM.cg <‐ phyloCorrelogram(navic, trait = "BM")
plot(IPSS.cg)
plot(random.cg)
plot(BM.cg)




The phylogenetic correlogram of *random* is flat and nonsignificant (Fig. 2A), while *BM* exhibits a positive autocorrelation for short lags (Fig. 2B). The correlogram of *IPSS* is a bit different with a strong positive autocorrelation for short lags and negative autocorrelation for medium lags (Fig. 2C). This is due to the clades structure of the signal: two closely related species belonging to the same clade tend to share similar trait values, but two adjacent clades are likely to differ strongly (Fig. 1).

Finally, we can compute local Moran's I for each species to detect hotspots of autocorrelation in *IPSS*. The following commands compute local Moran's I and represent them onto the phylogeny (Fig. 3). The *P*‐values are turned into colors to highlight hotspots. Here, we use a proximity matrix based on the number of nodes to ignore the effect of long terminal branches and focus on clades.



local.i <‐ lipaMoran(navic, trait = ”IPSS”,
 prox.phylo = “nNodes”, as.p4d = TRUE)
points.col <‐ lipaMoran(navic, trait = ”IPSS”,
 prox.phylo = “nNodes”)$p.value
points.col <‐ ifelse(points.col < 0.05, ”red”, ”black”)
dotplot.phylo4d(local.i, dot.col = points.col)




The LIPA analysis (Fig. 3) reveals significant local positive autocorrelation in two clades: the genus *Craticula* (including *Eolimna subminuscula*) with low values of sensitivity and the genus *Stauroneis* with high values of sensitivity.

## Conclusion

We have presented the phylosignal package and shown how it can be used to describe and analyze the phylogenetic signal in biological traits. The fact that phylosignal is integrated in the R ecosystem and uses the standard format phylo4d makes it interoperable with several other methods implemented in the R language. For example, users can complete these results with a phylogenetic principal component analysis (Jombart et al. 2010b) implemented in adephylo to detect combinations of traits that are phylogenetically autocorrelated. They can also use the tools implemented in ape to investigate evolutionary models through a generalized least squares approach (Paradis 2011). The combination of these tools will help to characterize the phylogenetic signal and to identify historical and ecological processes which drive patterns of trait values in the phylogeny.

## Conflict of Interest

None declared.
